# Association between functional outcomes and psychological variables in persons with spinal cord injury

**DOI:** 10.1038/s41598-023-50252-8

**Published:** 2023-12-28

**Authors:** Wonha Lee, SangHyup Jeong, Bum-Suk Lee, Jin-cheol Lim, Onyoo Kim

**Affiliations:** 1 Department of Physical Medicine and Rehabilitation, National Rehabilitation Center, 58, Samgaksan-ro, Gangbuk-gu, Seoul, 01022 Republic of Korea; 2 Department of Neuropsychiatry, National Rehabilitation Center, Seoul, Republic of Korea; 3 Department of Rehabilitation Medicine, Catholic Kwandong University, International St. Mary’s Hospital, Incheon, Korea; 4 Department of Education Measurement and Evaluation, Sungkyunkwan University, Seoul, Korea

**Keywords:** Health care, Medical research

## Abstract

We aimed to explore the association of functional outcomes with psychological variables, including depression, anxiety, sleep quality, and suicide risk, in persons with spinal cord injuries (SCIs). The secondary aim was to determine specific functions related to the psychological variables. This retrospective study included 259 persons with SCIs who were admitted to the Korean National Rehabilitation Center between 2019 and 2021. The participants were interviewed by a psychiatrist and completed questionnaires, including the Korean Beck Depression Inventory II (K-BDI-II), Korean Beck Anxiety Index, Insomnia Severity Index, and Mini International Neuropsychiatric Interview. To assess functional outcomes, the Spinal Cord Independence Measure III (SCIM III) and Walking Index for Spinal Cord Injury were determined by a physical therapist. The findings revealed a negative correlation of SCIM III subdivisions 1 and 3 with K-BDI-II. Specifically, feeding and mobility in bed and actions to prevent pressure injuries were functional factors associated with all four psychological variables. Our findings can guide clinicians to focus on improving functional independence and activities of daily living during the management of persons with SCI to prevent psychological consequences. Developing devices that aid in improving functional independence is crucial and may improve psychological problems in such individuals.

## Introduction

Spinal cord injury (SCI) is a lifelong condition that profoundly affects every aspect of life. Following SCI, patients experience serious physical disabilities, secondary complications, functional impairments, and psychological problems. Patients are placed in stressful environments that require adaptation, making them more susceptible to psychological disorders. The intensity and duration of psychosocial consequences can vary among patients and may last throughout their lives ^1^. Therefore, studying patients’ psychosocial problems is becoming increasingly important, and many systematic reviews and meta-analyses have evaluated the prevalence and factors associated with psychological disorders in persons with SCI.

The rate of psychological disorders may increase by 17%–25% in persons with SCI ^2^. Notably, depression, anxiety, posttraumatic stress disorder, insomnia, suicidal ideation, and substance abuse are more prevalent in persons with SCI than in the general population ^1^. For instance, in a meta-analysis by Williams et al. ^3^ that included 19 studies, the mean prevalence of depression among 35,676 participants was 22.2%. In another study by Le et al. ^4^ the estimated prevalence of anxiety based on self-reporting was 15–32%. Sleep disturbance is another frequently observed problem among patients that contributes to poor quality of life (QoL) and negative health outcomes, such as increased cardiovascular risk; furthermore, the absence of melatonin secretion can disrupt the circadian rhythm in persons with SCI ^5^. Albu et al. ^6^ previously showed that persons with SCI often report poor sleep quality, which can lead to reduced ability to participate in activities of daily living (ADLs) and decreased QoL, and that persons with SCI who were engaged in higher levels of physical activity reportedly experienced better sleep efficacy, reduced sleep disturbances, and shorter sleep latency. In addition, McCullumsmith et al. ^7^ reported that 13.3% of 2533 persons with SCI experienced suicidal ideation and that the estimated lifetime prevalence of suicide attempts was 7.4% in persons with SCI and > 4.6% in the general US population. A systematic literature review examining the prevalence of suicide among persons with SCI revealed that suicide accounted for 5.8%–11% of deaths in this population, and the presence of a psychiatric diagnosis emerged as a critical factor associated with suicide ^8^.

Many studies have focused on identifying predictive indicators of psychological consequences in persons with SCI. Several studies have investigated the association between functional outcomes and psychological variables. Mohammed et al. found that psychological variants, such as fear, anxiety, and depression, were negatively associated with physical function and performance ^9^. In addition, Kim et al. found that physical activity and mental health play crucial roles in persons with SCI. They observed that physically active individuals had lower levels of depression and anxiety and higher levels of social support ^10^. Another study reported a negative correlation of locomotor independence with symptoms of depression and anxiety in persons with SCI, regardless of etiological diagnosis or injury duration ^11^. While previous studies have shown an association between psychological variables and physical performance, including walking ability and ADLs, in persons with SCI, further studies are needed to identify the specific functional abilities associated with various psychological factors. Therefore, our study aimed to explore the relationship between functional outcomes and psychological factors in persons with SCI.

The primary objective of our study was to evaluate the association of demographic factors, SCI-related factors, and functional outcomes with the following psychological factors observed in persons with SCI: (1) depression, (2) anxiety, (3) sleep quality, and (4) suicide risk. Our secondary objective was to assess the specific items of the Spinal Cord Independence Measure III (SCIM III) that were associated with each psychological factor.

## Methods

### Participants

This retrospective study included persons with SCI admitted to the Korean National Rehabilitation Center between 2019 and 2021. The inclusion criteria were as follows: (1) age ≥ 19 years and (2) ability to understand and communicate normally. Patients who declined to participate or had a brain injury or cognitive disorder (Mini-Mental State Examination score < 24 points) that prevented them from understanding the psychiatric interview were excluded from the study.

### Study design

We collected general information, such as time since injury, type of SCI (completeness and level of injury), and etiology of the injury, from medical records. Personal details, such as age, sex, religion, caregiver status (family or paid caregiver), marital status, and employment status, as well as presence of spasticity and pain were collected through interviews conducted by a psychiatrist.

Functional outcomes were assessed using the SCIM III and the Walking Index for Spinal Cord Injury II (WISCI II), which were measured by a physical therapist at the time of admission. The SCIM III assesses the level of independence in persons with SCI based on 19 ADLs divided into three domains: (1) self-care (eating, grooming, bathing, and dressing), (2) respiration and sphincter management, and (3) mobility (bed-related tasks, transfer, and ambulation indoors and outdoors). The scale ranged from 0 to 100 points, with 0 representing complete dependence and 100 representing complete independence. The scale is distributed as follows: self-care (0–20 points), respiration and sphincter management (0–40 points), and mobility (0–40 points). The SCIM III has shown high validity, supported by a strong correlation with Functional Independence Measure (FIM) scores (Pearson correlation coefficient of 0.790, p < 0.01), and reliability, supported by intra-class correlation coefficient values exceeding 0.94 for both the total SCIM III and SCIM subscales ^12^. The SCIM III is a highly valid and reliable tool for determining the level of independence in persons with SCI ^12,13^. Ambulation function was assessed using WISCI II, which evaluates the amount of physical assistance and the number of assistive devices required to walk for 10 m. The scale score ranges from 0 to 20 points, with 0 indicating inability to stand or participate in assisted walking and 20 indicating ability to demonstrate the highest level of walking performance (i.e., unassisted walking, without any devices, for 10 m) ^14^.

Neurological examinations were performed by a physical therapist at the time of admission, according to the International Standards for the Neurological Classification of Spinal Cord Injury ^15^. Upper extremity motor sub-scores (UEMS) and lower extremity motor sub-scores (LEMS) were evaluated as the sum of the key muscles of each extremity’s Manual Muscle Test (MMT) scores.

Psychological variables, including depression, anxiety, sleep quality, and suicide risk, were assessed by a psychiatrist. Depressive symptoms were evaluated using the Korean Beck Depression Inventory II (K-BDI-II). The K-BDI-II contains 21 items scored on a three-point scale that measures depressive symptoms, including sadness, guilt, failure, dissatisfaction, irritability, hopelessness, punishment, and suicidal ideation, and physical symptoms, including weight loss, fatigue, and sexual disinterest. The maximum possible score is 63 points, with higher scores indicating higher levels of depression. A cutoff score of 18 points has been suggested to achieve 85% sensitivity ^16^. The Korean Beck Anxiety Index (K-BAI) was used to assess anxiety. This index consists of 21 items evaluated on a 4-point Likert scale ranging from 0 to 4 points, measuring the somatic and cognitive symptoms of anxiety. Based on Oh et al.’s investigation, the optimal K-BAI cutoff score, where high sensitivity and specificity can be achieved, is 8 points ^17^. Sleep quality was assessed using the Insomnia Severity Index (ISI), which consists of seven questions measuring the severity of symptoms, satisfaction with sleep patterns, and overall level of distress caused by sleep problems ^18^. Finally, suicide risk and severity were measured using the Mini International Neuropsychiatric Interview (MINI). The MINI consists of six questions, including suicidal thoughts and behaviors, to define four levels of suicide risk: no risk, low risk, average risk, and high risk ^19^.

### Statistical analysis

Data were analyzed using SPSS, Version 27.0 (IBM Corp., Armonk, NY, USA). Continuous data are described using means and standard deviations (SD), and categorical data are presented using percentages and proportions. Student’s t-test was used to compare continuous data between the two groups, including demographic factors (sex, religion, caregiver, marital status, and employment status), SCI factors (American Spinal Injury Association Impairment Scale [AIS] score and level of injury), and psychological variables (K-BDI-II, K-BAI, ISI, and MINI). Pearson’s correlation was used to compare differences between age and psychological variables. It was also used to identify the relationships between functional outcomes (SCIM III, WISCI II level, UEMS, and LEMS) and psychological variables. Statistical significance was set at *p* < 0.05. Missing data were handled through listwise deletion, followed by correlation analyses for each psychological factor. Regarding assumption of normality, we followed the central limit theorem, which allowed us to assume normality in our data, irrespective of the data distribution, as our sample size was sufficiently large (> 30) ^20,21^. Consequently, parametric procedures were employed in our analysis.

### Ethics declaration

Upon admission, informed consent was obtained from all individuals for the use of their psychological and physical examination results. This study was approved by the Ethics Committee at the National Rehabilitation Center, Korea (NRC-2022-01-005) and performed in accordance with relevant regulations and guidelines.

## Results

### Demographics and clinical characteristics of the participants

The demographic and clinical characteristics of the 259 participants (196 men and 63 women) with SCI are shown in Table 1. The mean age was 49.2 years; 144 participants were married and 92 were unmarried (including unmarried, divorced, bereaved, or estranged). Of the 259 participants, 171 had incomplete SCI (AIS B, C, and D), and 80 had complete SCI (AIS A). Overall, 140 tetraplegic (55.1%) and 114 paraplegic (44.9%) patients were enrolled, and the majority of the participants were diagnosed with traumatic injury (70.2%). The average scores for the psychological variants, represented by K-BDI-II, K-BAI, ISI, and MINI, were 15.0, 8.6, 4.8, and 1.9 points, respectively.

**Table 1:** Demographics and clinical characteristics of the participants.

Variables	Category	Full Sample N (%)	Mean (SD) or N (%)
Age	–	259 (100.0)	49.2 (16.5)
Sex	Male/female	259 (100.0)	196 (75.7)/63 (24.3)
Religion	Yes/no	239 (92.3)	138 (53.3)/101 (39.0)
Caregiver	Family/paid caregiver	256 (98.8)	152 (58.7)/104 (40.2)
Marital status	Married/unmarried	236 (91.1)	144 (55.6)/92 (35.5)
Employment status	Yes/no	235 (90.7)	85 (32.8)/150 (57.9)
Time duration from the injury (days)	–	251 (96.9)	716.7 (1867.8)
AIS	A/B, C, D	254 (98.1)	80 (31.9)/171 (68.1)
Level of injury	Tetraplegia/paraplegia	252 (97.3)	140 (55.1)/114 (44.0)
Etiology	Traumatic/non-traumatic	259 (100.0)	177 (70.2)/75 (29.8)
Spasticity	Yes/no	259 (100.0)	79 (30.5)/180 (69.5)
Pain	Yes/no	259 (100.0)	180 (69.5)/79 (30.5)
UEMS	–	259 (100.0)	35.9 (15.7)
LEMS	–	259 (100.0)	16.4 (16.5)
Total MMT	–	259 (100.0)	52.5 (24.5)
WISCI II level	–	259 (100.0)	8.9 (6.9)
Depression (K-BDI-II)	–	240 (96.9)	15.0 (10.9)
Anxiety (K-BAI)	–	176 (68.0)	8.6 (9.0)
Sleep Quality (ISI)	–	211 (81.5)	4.8 (4.9)
Suicidal risk (MINI)	–	217 (83.8)	1.9 (3.2)

Unmarried (unmarried, divorce, bereavement, or estrangement); Religion (Christian, Catholic, Buddhist).

AIS, American Spinal Injury Association Impairment Scale; ISI, Insomnia Severity Index; K-BAI, Korean Beck Anxiety Index; K-BDI-II, Korean Beck Depression Inventory II; LEMS, Lower extremity motor sub-scores; MINI, Mini International Neuropsychiatric Interview; MMT, Manual Muscle Test; N, number; SD, standard deviation; UEMS, upper extremity motor sub-scores; WISCI II, Walking Index for Spinal Cord Injury II.

### Comparison of demographics and psychological factors (depression, anxiety, sleep quality, and suicide risk)

The results revealed no association of the patients’ demographic and injury characteristics with psychological variables (Table 2). Further, no significant association was noted between the severity of SCIs, as categorized on the AIS (AIS A, B, C, or D), and psychological factors. Contrastingly, a significant association was identified between marital status and suicide risk, as indicated by the MINI score. Specifically, the average MINI score was 1.5 in the married group and 2.8 in the unmarried group, indicating that the unmarried group had an increased suicide risk *(p* < *0.023)*. Another statistically significant difference was found between age and suicide risk, in which age was negatively correlated with suicide risk *(p* < *0.024)*. Other demographic and injury-related variables did not have a statistically significant association with the psychological variants.

**Table 2:** Comparison of demographics and psychological factors (depression, anxiety, sleep quality, and suicide risk).

Variables	Depression (K-BDI-II)		Anxiety (K-BAI)		Sleep quality (ISI)		Suicide risk (MINI)	
	M (SD) or r	*p*	M (SD) or r	*p*	M (SD) or r	*p*	M (SD) or r	*p*
Sex^a^								
Male	14.3 (9.9)	.143	7.9 (7.8)	.133	4.5 (4.6)	.102	1.7 (3.1)	.205
Female	17.2 (13.3)		10.8 (11.9)		5.8 (5.7)		2.4 (3.6)	
Religion^a^								
Yes	13.7 (10.3)	.065	7.7 (7.2)	.177	4.8 (5.0)	.921	1.7 (2.8)	.685
No	16.5 (11.8)		9.8 (11.3)		4.7 (4.8)		1.9 (3.4)	
Care giver^a^								
Family	14.6 (10.6)	.577	8.6 (8.3)	.875	4.8 (4.8)	.928	1.6 (2.7)	.155
Paid caregiver	15.4 (11.1)		8.4 (10.0)		4.7 (5.1)		2.3 (3.8)	
Marital status^a^								
Married	15.0 (10.9)	.942	8.0 (7.0)	.353	4.5 (4.4)	.380	1.5 (2.9)	**.023**
Unmarried	15.1 (10.7)		9.3 (10.0)		5.2 (5.8)		2.8 (3.8)	
Employment status^a^								
Yes	13.8 (10.7)	.189	8.3 (7.8)	.840	4.2 (4.6)	.231	1.7 (3.3)	.337
No	15.8 (11.0)		8.6 (8.6)		5.1 (5.1)		2.1 (3.3)	
AIS^a^								
A	15.9 (11.4)	.376	8.0 (9.9)	.654	5.0 (4.7)	.649	1.8 (3.2)	.787
B, C, D	14.6 (10.6)		8.7 (8.8)		4.7 (5.1)		1.9 (3.3)	
Level of injury^a^								
Tetraplegia	15.9 (11.7)	.185	9.8 (11.5)	.066	5.0 (4.9)	.473	2.2 (3.6)	.150
Paraplegia	14.0 (9.9)		7.3 (5.0)		4.5 (5.0)		1.5 (2.6)	
Etiology^a^								
Traumatic	15.2 (10.9)	.434	8.7 (9.9)	.719	4.7 (4.8)	.619	1.8 (3.3)	.572
Non-traumatic	14.0 (10.8)		8.1 (6.8)		5.1 (5.2)		2.1 (3.1)	
Spasticity^a^								
Yes	16.7 (11.6)	.125	9.3 (8.2)	.474	5.0 (5.1)	.670	2.4 (3.7)	.111
No	14.3 (10.5)		8.3 (9.3)		4.7 (4.8)		1.6 (3.0)	
Pain^a^								
Yes	15.1 (10.9)	.835	8.9 (8.6)	.434	5.0 (4.9)	.415	1.8 (2.7)	.650
No	14.8 (10.9)		7.7 (9.9)		4.4 (4.9)		2.0 (4.2)	
Age^b^	0.07	.257	0.04	.645	0.08	.229	− 0.153	**.024**
Time duration from the injury^b^	0.12	.069	0.13	.079	0.06	.421	0.043	.529

ISI, Insomnia Severity Index; K-BAI, Korean Beck Anxiety Index; K-BDI-II, Korean Beck Depression Inventory II; MINI, Mini International Neuropsychiatric Interview; SD, standard deviation.

Tests: ^a^Student’s *t*-test, ^b^Pearson’s *r* correlation. Bold values: *p* < .05.

### Correlation between functional outcomes and psychological variables in persons with SCI

The correlation between the functional outcomes and psychological variables in persons with SCI is shown in Table 3. The SCIM III is divided into three parts: subdivision 1, self-care (feeding, bathing, dressing, and grooming); subdivision 2, respiration and sphincter management (bladder and bowel); and subdivision 3, transfer and ambulation (bed mobility, ability to move to prevent pressure injuries, transfers from bed to wheelchair or wheelchair to toilet or car, and indoor and outdoor mobility). SCIM III, UEMS, and total MMT scores were negatively correlated with the K-BDI-II. The Pearson correlation coefficient of each functional outcome ranged from − 0.16 to − 0.20, indicating similar levels of correlation. SCIM III subdivision 1 showed a correlation with all psychological factors (K-BDI-II *p* < 0.003, K-BAI *p* < 0.02, ISI *p* < 0.012, and MINI *p* < 0.003), whereas SCIM III subdivision 3 and SCIM III total were only related to depression, insomnia, and suicide risk. The WISCI II was not correlated with any psychological factor. Taken together, total SCIM III, SCIM III subdivisions 1 and 3, and UEMS scores were negatively correlated with psychological factors.

**Table 3:** The correlation between functional outcomes and psychological variables in persons with SCI.

Variables	Depression (K-BDI-II)		Anxiety (K-BAI)		Sleep quality (ISI)		Suicide risk (MINI)	
	*r*	*p*	*r*	*p*	*r*	*p*	*r*	*p*
SCIM sub1	− 0.192	**.003**	− 0.175	**.020**	− 0.173	**.012**	− 0.210	**.003**
SCIM sub2	− 0.160	**.013**	− 0.057	.450	− 0.076	.271	− 0.153	**.034**
SCIM sub3	− 0.190	**.003**	− 0.144	.056	− 0.178	**.009**	− 0.213	**.003**
SCIM total	− 0.203	**.002**	− 0.133	.077	− 0.157	**.022**	− 0.216	**.003**
WISCI II level	− 0.091	.161	0.002	.984	− 0.124	.073	− 0.105	.147
UEMS	− 0.161	**.012**	− 0.169	**.025**	− 0.133	.054	− 0.171	**.017**
LEMS	− 0.088	.172	0.045	.552	0.002	.973	− 0.051	.482
Total MMT	− 0.162	**.012**	− 0.075	.324	− 0.083	.231	− 0.142	**.048**

Tests: Pearson correlation; Pearson partial correlations between variables related to suicide risk, with adjustment for age and marital status. Bold values: *p* < .05. Depression (df = 238), Anxiety (df = 174), Sleep quality (df = 209), Suicide risk (df = 191).

ISI, Insomnia Severity Index; K-BAI, Korean Beck Anxiety Index; K-BDI-II, Korean Beck Depression Inventory II; SCIM III, Spinal Cord Independence Measure III; LEMS, lower extremity motor sub-scores; MMT, Manual Muscle Test; sub, subdivision; MINI, Mini International Neuropsychiatric Interview; SCI, spinal cord injury; UEMS, upper extremity motor sub-scores; WISCI II, Walking Index for Spinal Cord Injury.

### Relationship between functional compositions of each subdivision of SCIM III and psychological variables

The correlation between SCIM III subdivisions 1 and 3 and most psychological variables (except anxiety) was statistically significant (SCIM III subdivision 1; K-BDI-II [*p* = 0.006], K-BAI [*p* = 0*.*003], ISI [*p* = 0*.*034], and MINI [*p* = 0*.*01]/SCIM III subdivision 3; K-BDI-II [*p* < 0*.*001], K-BAI [*p* = 0*.*006], ISI [*p* = 0*.*014] and MINI [*p* < 0.001]). The functional compositions of SCIM III subdivisions 1 and 3 were analyzed to determine which specific functional abilities were related to psychological factors. The relationships between the functional compositions of SCIM III subdivisions 1 and 3 and psychological variables are shown in Table 4. Most sub-factors from SCIM III subdivision 1 were correlated with depression, and out of SCIM III subdivision 1, eating was correlated with all four psychological factors. SCIM III subdivision 3 sub-factors consisting of ambulation ability and positional changes to prevent pressure injury, transfer from bed to wheelchair and wheelchair to toilet, as well as indoor ambulation were correlated with depression, insomnia, and suicide risk. Changing positions to prevent pressure injuries was associated with all four psychological factors.

**Table 4:** The relationship between functional compositions of each SCIM III subdivision and psychological variables.

Variables	Depression (K-BDI-II)		Anxiety (K-BAI)		Sleep quality (ISI)		Suicide risk (MINI)	
	*r*	*p*	*r*	*p*	*r*	*p*	*r*	*p*
SCIM III subdivision 1								
Feeding	− 0.177	**.006**	− 0.223	**.003**	− 0.146	**.034**	− 0.186	**.010**
Bathing (upper body)	− 0.118	.067	− 0.104	.168	− 0.147	**.033**	− 0.210	**.003**
Bathing (lower body)	− 0.174	**.007**	− 0.094	.213	− 0.213	**.002**	− 0.202	**.005**
Dressing (upper body)	− 0.153	**.018**	− 0.159	**.035**	− 0.108	.116	− 0.205	**.004**
Dressing (lower body)	− 0.201	**.002**	− 0.108	.152	− 0.215	**.002**	− 0.213	**.003**
Grooming	− 0.165	**.011**	− 0.174	**.021**	− 0.100	.149	− 0.160	**.026**
SCIM III subdivision 3								
Mobility in bed and action to prevent pressure injuries	− 0.226	** < .001**	− 0.205	**.006**	− 0.169	**.014**	− 0.240	** < .001**
Transfers: bed-wheelchair	− 0.163	**.012**	− 0.130	.086	− 0.178	**.010**	− 0.240	** < .001**
Transfers: wheelchair-toilet-tub	− 0.133	**.039**	− 0.107	.158	− 0.146	**.035**	− 0.215	**.003**
Mobility indoors	− 0.121	.060	− 0.075	.320	− 0.154	**.026**	− 0.171	**.017**
Mobility for moderate distances (10–100 m)	− 0.167	**.010**	− 0.137	.069	− 0.185	**.007**	− 0.170	**.018**
Mobility outdoors (> 100 m)	− 0.115	.075	− 0.100	.187	− 0.079	.252	− 0.113	.118
Stair management	− 0.133	**.040**	− 0.029	.700	− 0.054	.435	− 0.063	.381
Transfers: wheelchair-car	− 0.093	.152	− 0.016	.837	− 0.128	.063	− 0.092	.201
Transfers: ground-wheelchair	− 0.116	.073	− 0.056	.461	− 0.142	**.039**	− 0.108	.134

Tests: Pearson correlation; Pearson partial correlations between variables related to suicide risk, with adjustment for age and marital status. Bold values: *p* < .05. Depression (df = 238), Anxiety (df = 174), Sleep quality (df = 209), Suicide risk (df = 191).

ISI, Insomnia Severity Index; K-BAI, Korean Beck Anxiety Index; K-BDI-II, Korean Beck Depression Inventory II; SCIM III, Spinal Cord Independence Measure III; MINI, Mini International Neuropsychiatric Interview.

## Discussion

This study investigated the relationship between functional performance and psychological factors, including depression, anxiety, sleep quality, and suicide risk, in persons with SCI. The results showed that the total SCIM III, SCIM III subdivision 1 (self-care), subdivision 3 (transfer and ambulation), and UEMS were negatively correlated with psychological variables. Specifically, the functions most significantly related to all psychological variables were feeding and changing positions to prevent pressure injuries.

### Functional outcomes were significantly correlated with psychological factors

Functional outcomes were strongly related to psychological factors rather than to various demographic and SCI-related factors. All items of the SCIM III were related to depression. Moreover, SCIM III subdivision 1 (self-care) and subdivision 3 (ambulation/transfer) were related to anxiety, sleep quality, and suicide risk. Walking function, as shown by the WISCI II, was not related to psychological factors. In a similar study by Qasheesh et al*.*, there was a strong negative correlation between functional outcomes and psychological variants, including fear, anxiety, and depression, with depression as the variant with the strongest correlation ^8^. In contrast, another study on 36 persons with SCI who had been injured within 6 months before the study reported a higher rate of depression and an association between SCI-related factors, such as motor-complete injury, and depression ^22^. In the same study, functional independence assessment tools, including the Modified Barthel Index, FIM, and SCIM, were used, and depression and functional outcomes were found to be unrelated. This discrepancy may be explained by the fact that the second study included patients who were injured within 6 months, making it too early to evaluate functional outcomes ^22^.

### All functional outcomes were strongly associated with psychological factors, where eating and postural changes had the most significant association

Our study investigated which functional outcomes among all the items of the SCIM III were strongly related to psychological factors. Feeding, bed mobility, and postural changes to prevent pressure injuries were key functional outcomes related to all four psychological variables and may be clinically related to the QoL of persons with SCI.

SCIM III subdivision 1 consists of self-care activities, such as feeding, bathing, dressing, and grooming, all of which were related to depression. Moreover, feeding was related to all four psychological factors. Other studies have investigated functional priorities for achieving a high QoL in persons with SCI. Among the five functional priorities (arm/hand use, walking, bladder/bowel control, sexual function, and presence of pain), arm/hand function had a 44%–76% higher preference than other functions, and walking was the least important functional priority. Restrictions on arm/hand function limit the ability to live independently and perform the most basic activities of our lives. Further studies have highlighted the correlation between upper limb function and various psychological factors. In this respect, Armstrong et al*.* investigated the association between different psychological factors in patients with upper extremity limb loss and revealed a significant association between psychological distress, including depression and posttraumatic stress disorder, and upper extremity limb loss, irrespective of severity and etiology ^23^. Therefore, the rehabilitation of arm and hand function possesses immense potential for enhancing QoL ^24^. This is consistent with our finding that SCIM III subdivision 1 (self-care) was mostly related to various psychological variables.

SCIM III subdivision 3 consists of transfer and ambulation tasks. Transfers can include transfers from the bed, wheelchair, toilet, tub, car, or ground. Ambulation refers to indoor or outdoor activities, with or without a wheelchair or other aid devices. Our study demonstrated that independent bed mobility and transfer are important functional abilities in persons with SCI. In particular, bed mobility and transfer are critical for preventing pressure injuries. Pressure injury, a serious complication in persons with SCI, lowers QoL and rehabilitation outcomes, resulting in psychological, physiological, and economic burdens. Charalambous et al. found that pressure injuries can have a detrimental effect on stress levels resulting from pain, odor, and restrictions in daily activities ^25^. In addition, Flett et al*.* reported that the FIM score of bed/chair transfer was significantly related to the risk of developing pressure injury ^26^. These results emphasize that, irrespective of the ambulatory function, the ability to mobilize in bed and transfer independently, thereby preventing pressure injury, can have a positive psychological effect on persons with SCI.

### Functional outcomes are more important than demographic and clinical factors

Previous studies have examined various demographic and SCI-related factors that may affect psychological variables after SCI. However, there is no consensus wherein demographic factors are related to psychological variables. Khazaeipour et al*.* found that depression was associated with female sex, tetraplegia, low educational level, and having a family member as a caregiver ^27^. However, in another study examining 849 patients with depression and SCI, Bombardier et al*.* concluded that demographic factors, such as sex and education level, as well as injury-related factors, such as level and severity of injury, were not significantly associated with depression and anxiety ^28^. Our study revealed that marital status and age were associated with suicide risk, whereas other demographic factors were not significantly related to psychological variables. Additionally, while lower levels of cord injury were associated with greater voluntary movement and expectations of independence, the level and extent of injury were not related to psychological variables. Instead, our study found that the individual’s functional outcomes were strongly related to psychological factors. Therefore, rehabilitation strategies should be developed based on the individual’s physical performance, and the functional independence of the individual must be reinforced while providing optimal psychological support.

### Future plans

Based on the results of our study, restrictions on performing ADLs were negatively correlated with psychological problems, emphasizing the importance of regaining functional independence as a crucial factor for enhancing QoL. Assistive technologies have been developed to alleviate limitations in patient independence. Robotic technology is being developed worldwide to promote the independent living of elderly and disabled patients. For example, an intelligent bed robot system was developed to measure pressure and help detect proper posture to prevent pressure injury ^29^. Devices, such as dynamic arm support or robotic arms (ROSE, iArm), are commercially available to maximize the independence of the arm and hand functions to aid in grabbing and moving objects ^30^. The development of such devices can functionally improve the independence of persons with SCI and possibly alleviate their psychological problems.

As our study highlights the importance of functional independence in persons with SCI, clinicians can elaborate on the patients’ rehabilitation plans and set specific goals for related treatments in the rehabilitation setting. They should focus on ADLs and functional outcomes, and major goals should include improving the individual’s functional independence, enhancing their QoL, and avoiding possible psychological distress.

### Limitations and strengths

This study had some limitations. First, it was retrospective in nature, and selection bias or confounding variables may have been present; thus, it was difficult to establish causality, and we were only able to examine the association between psychological and functional factors. In addition, our study only included participants in rehabilitation settings and those with injuries for a mean duration of 2 years. As a result, selection bias might have been elicited, as patients who did not receive rehabilitation or had injuries lasting > 2 years were excluded. Thus, our results may not represent the entire SCI population.

However, this study is significant as, to the best of our knowledge, it is the first to evaluate specific functional abilities concerning various psychological factors in persons with SCI. Our study addressed various psychological factors using widely used and validated questionnaires and focused on functional outcomes rather than demographic factors. Another strength of our study is the large number of participants. Our findings can help guide clinicians in identifying patients at a higher risk of experiencing psychological consequences.

## Conclusion

Functional independence in persons with SCI was associated with various psychological factors, including depression, anxiety, sleep disorders, and suicide risk. Independent feeding and the ability to move independently to prevent pressure injuries were the most important functional abilities related to psychological factors. Ambulation ability, as assessed by the WISCI II scores, was not significantly associated with psychological variables. Therefore, improving functional independence should be an important part of rehabilitation to help reduce psychological distress and enhance the overall QoL of patients.

## Data Availability

The datasets generated during and/or analyzed during the current study are available from the corresponding author upon reasonable request.
